# Applying Machine Learning Technologies Based on Historical Activity Features for Multi-Resident Activity Recognition

**DOI:** 10.3390/s21072520

**Published:** 2021-04-04

**Authors:** Jia-Ming Liang, Ping-Lin Chung, Yi-Jyun Ye, Shashank Mishra

**Affiliations:** 1 Department of Electrical Engineering, National University of Tainan, Tainan 70005, Taiwan; d10982003@stumail.nutn.edu.tw; 2 Department of Computer Science and Information Engineering, Chang Gung University, Taoyuan 33302, Taiwan; M0829005@cgu.edu.tw (P.-L.C.); M0629011@cgu.edu.tw (Y.-J.Y.)

**Keywords:** ambient assisted living, multi-person activity recognition, machine learning, deep learning

## Abstract

Due to the aging population, home care for the elderly has become very important. Currently, there are many studies focusing on the deployment of various sensors in the house to recognize the home activities of the elderly, especially for the elderly living alone. Through these, we can detect the home situation of the single person and ensure his/her living safety. However, the living environment of the elderly includes, not only the person living alone, but also multiple people living together. By applying the traditional methods for a multi-resident environment, the “individual” activities of each person could not be accurately identified. This resulted in an inability to distinguish which person was involved in what activities, and thus, failed to provide personal care. Therefore, this research tries to investigate how to recognize home activities in multi-resident living environments, in order to accurately distinguish the association between residents and home activities. Specifically, we propose to use the special characteristics of historical activity of residents in a multi-person environment, including activity interaction, activity frequency, activity period length, and residential behaviors, and then apply a suite of machine learning methods to train and test. Five traditional models of supervised learning and two deep learning methods are explored to tackle this problem. Through the experiments with real datasets, the proposed methods were found to achieve higher precision, recall and accuracy with less training time. The best accuracy can reach up to 91% and 95%, by J48DT, and LSTM, respectively, in different living environments.

## 1. Introduction

A smart home is a promising technology for elderly care, which can be realized by *Internet of things (IoT)*, to improve the quality of life of the elderly, prevent and detect their accidents, and enable the elderly to live in a safe and comfortable environment [1,2,3,4]. As the number of elderly people is increasing, *Ambient Assistant Living (AAL)* [5,6] has been fully applied for home care. In the AAL environment, by deploying multiple sensors to detect home activities, it can achieve elderly care, such as accident detection, emergency medical rescue, and remote assistance [7,8]. However, existing researches mainly focused on vision based activity recognition [9,10,11], body action identification [12,13,14,15], or single-person activity recognition [16]. This may pose face privacy concerns, or be difficult to associate body action with home activity, or neglect the elderly living together with spouse or even relatives and/or friends [17,18,19], respectively. Once single-person activity recognition is applied to the multi-person environment, it will fail to identify different residents’ activities, and thus, cannot provide personal care for the elderly. This becomes an important issue and motivates us to study this problem.

Therefore, this paper tries to address such activity recognition problem in the multi-resident environment and proposes to explore a suit of machine learning models to tackle this problem. Specifically, we investigate and implement five traditional machine learning methods (including *Support Vector Machine*, *K-Nearest Neighbor*, *Multi-Layer Perceptron*, *J48 Decision Tree*, *Random Forest*) and two deep learning techniques (including *Recurrent Neural Networks* and *Long Short-Term Memory*) for modeling and testing, and then apply six special historical activity features with multi-label methods [20] to evaluate their benefits based on the confusion matrix and 10-Fold Cross-Validation. Note that the deep learning models have the nature to handle time series features, which are fitting with the characteristics of sequential home activities. However, these deep learning based techniques may have higher complexity cost. We investigate them with the traditional well-known learning models together. The extensive findings provide important observations with respect to traditional methods and deep learning techniques. These methods can achieve better performances in terms of precision, recall and accuracy in different scenarios, respectively.

The contributions of this paper are three-fold. First, this is a complete work to address the problem of multi-resident home activity recognition. Contrary to the literature, this work aims to identify ‘which resident’ activates ‘what activity’, that is more difficult than traditional recognition problems. Second, we first design six special characteristics based on residents’ historical behaviors and activity dependency, and then propose to leverage a suit of machine learning models, including five traditional supervised learning and two new deep learning models, to investigate and conclude their effectiveness, efficiency, and complexity. Third, we evaluate the performance of the proposed schemes and verify them based on a public and well known dataset (i.e., ARAS dataset), which is generally acknowledged and can make our results more authoritative and recognized. These results also reveal that J48DT and LSTM methods can achieve better precision, recall and accuracy in different environments, which can provide valuable experiences for the system developers of elderly personal care in the future.

The rest of this paper is organized as follows. Related work is discussed in Section 2. We define our problem in Section 3. Section 4 presents our proposed method. Performance evaluations are shown in Section 5. Conclusions are drawn in Section 6.

## 2. Related Work

In the literature, due to the increase of the elderly population, home activity recognition has been widely used in smart homes. Among them, the *AAL (ambient assisted living)* platform [21,22] are designed to achieve home care, such as detecting abnormal activities of the elderly and even for the elderly with dementia [23]. Traditionally, *CCTV (closed-circuit television)* is a common way for monitoring and recognizing activity [9,10]. However, they face privacy concerns. Therefore, the research [11] proposes a privacy-preserving and computationally efficient framework. Whereas the work [12] explores data acquisition, transmission and data encryption to mitigate residential privacy issues. In addition, the references, [13,14,15,16], further leverage sensors to detect activities and reduce the leakage of image privacy. Specifically, the studies [13,14] use acceleration sensors to identify body activities. While, the research [15], uses multilevel classification to achieve activity identification. However, the above studies require to wear sensors over a long period of time, which would make residents uncomfortable, and thus, disassemble them, resulting in losing results. Then, the work, [16], combines multiple WiFi signals and exploits signal strength information to achieve activity recognition. However, it only focuses on single-person activity recognition.

Based on the above results, it motivates us to study the home activity recognition in the multi-resident environment by using non-intrusive sensing devices, such as non-imaging and non-wearing technologies to avoid privacy concerns. We summarize the above literature in Table 1. 

## 3. Problem Definition

In this paper, we focus on the most common home activities, which are shown in Table 2. We assume that each user’s historical activities in the past *N* days have been known, as shown in Table 3, where *A* represents the activity number, *T* represents the activity time, and *U* represents the user who performed the activity. Such data is recorded by an activity structure of {(*A_i_*, *T_i_*, *U_i_*), *i* = 1, …, *N*}, where *i* is the *i-th* day. Our goal is to ask how to correctly match the *unknown activity sequence A_x_* and the *unknown user sequence U_x_* based on the given activity list {(*A_i_*, *T_i_*, *U_i_*), *i* = 1…, *N*} so as to provide personal care. The notations used in this paper are summarized in Table 4.

## 4. The Proposed Methods

In this work, we study the activity recognition issue in multi-resident environments and propose various machine learning models based on the activity-dependent characteristics and historical activity behaviors of the residents, thereby improving the accuracy of the activity recognition. Specifically, our solution is divided into two stages. The first stage is data collection and pre-processing, which detects whether the data is abnormal or missing, and then performs the numerical conversion. The second stage is to implement a suit of machine learning models, and then performs training and testing. Finally, we make experiments and analyze the benefit to obtain the better classification results. The details are described as follows.

### 4.1. Data Collection and Preprocessing

We use the ARAS dataset [24] in this work, which collects real data from two houses with multiple residents, and applies a non-invasive detection method by deploying multiple sensors to recognize home activities, including pressure sensor, temperature sensor, and distance sensor, and infrared receiver, etc. This dataset records 30 days of home activities. The detailed attributes of the dataset are shown in Table 5.

For the ARAS dataset, we first performed multi-label merging [20] for the 27 activities identified in the dataset, combining similar activity types, such as preparing breakfast, preparing lunch, and preparing dinner, into “cooking” and use “eating” to represent having breakfast, having lunch, and having dinner to enhance the nature of the activity. The combined results are shown in Table 6 and Table 7.

- Data Preprocessing

**Figure 1:**
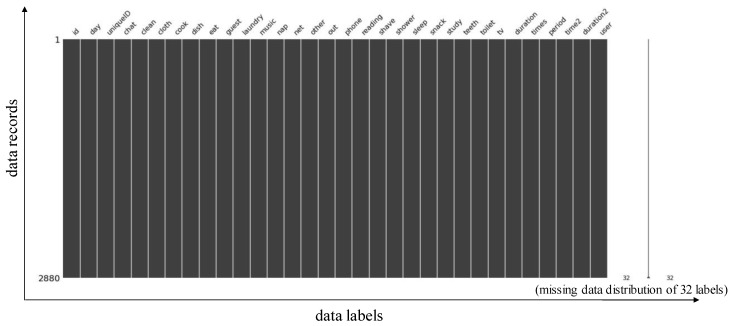
Data visualization.

Next, we visualize the data to check whether the data is abnormal or missing. In addition, we test it based on the “missingno” package (built in Python). The results are shown in Figure 1. As can be seen, there is no white dashed line in the black bar-graph (i.e., missing value), which means that the dataset is complete and no compensation is required. Note that the left vertical axis means the number of data records, the horizontal axis is the data label, and the right side shows the missing data distribution. Since the dataset has no missing data, this distribution has no variation and only shows the number of total data labels (i.e., 32). 

Then, through *One-Hot Encoding*, the non-numeric fields are converted into numerical values. Since there is no difference in the order of the content in each line, it will not be converted into numerical values with difference size, but converted into dummy variables, as shown in Table 8. Note that the two residents are marked as “user_1_” and “user_2_” in the right part of Table 8 (in the red block). The ID shows “2880” means that it has 2880 records of activity data after data processing.

- Standardization of Features

In order to prevent the values of specific feature in the dataset from affecting the classification results, we use *Z-Score Normalization* [25] as the method, shown in the following:

(1) $$New\;value=\;\frac{x-\mu }{\sigma }.$$

This method normalizes the original data to a value with a mean value of 0 and a variance of 1, where *x* represents the value before normalization, μ represents the feature average, and  σ  represents the feature standard deviation.

- Feature Selection

Here, we use the *filtering method* [26], which scores each feature value according to the divergence and correlation index of the feature, and determines the suitable feature by setting the scoring threshold or the number of selected thresholds. 

Through the above processes, the features of the home activities are filtered out: 1. Activity sequence of a day (labeled by *uniqueID*), 2. Activity frequency of a day (labeled by *times*), 3. Activity duration (in hours, labeled by *duration*), 4. Activity occurrence interval (labeled by *period*, which is represented as: early ‘1’, mid ‘2’ or late ‘3’), 5. Activity occurrence times in a day (labeled by *times2*), 6. Ratio of individual activity over total activities (labeled by *ratio*). The examples of filtered activity features are shown in Table 9. 

### 4.2. Machine Learning Methods

In the second stage, we implement a suit of machine learning methods to explore the correctness of multi-resident activity recognition, including five traditional machine learning methods: *Support Vector Machine (SVM)*, *K-Nearest Neighbor (KNN)*, *Multi-Layer Perceptron (MLP)*, *J48 Decision Tree*, *Random Forest (RF),* and two deep learning models: *Recurrent Neural Networks (RNN)* and *Long Short-Term Memory (LSTM)*. The details are described as follows.

- Support Vector Machine (SVM)

*Support Vector Machine (SVM)* [27] is a supervised machine learning method. It was originally used for binary classification, but has now been extended to statistical classification and regression analysis. The principle involves using the function to upgrade the data from the original dimension to the high-dimensional feature space, and use the optimization tool in the feature space to find a hyper-plane that can separate the data into two categories. This hyper-plane is the classification boundary. Therefore, a good classification boundary should be as far away as possible from the nearest training data point, which can reduce the probability of judgment errors. 

Note that we implemented the multi-classification SVM based on the *One-Against-One method* [28], which selects certain two categories of data from the multi-category data, and repeats the operation until all the category combinations have their corresponding SVMs. Finally, there will be $C_{2}^{T}\;$ (T-class classification) SVM models. When there is a new piece of data to be predicted, $C_{2}^{T}$ SVMs will be thrown into it. Each SVM will classify this data into a certain category like voting, then record +1 for this category, and finally judge the category with the maximum number of votes. Then, it can predict the data belonging to that category.

- K-Nearest Neighbor (KNN)

*K-Nearest Neighbor (KNN)* [29] is one of the simplest supervised classification method. The concept of the method is to determine one’s own category based on the neighbor categories that are close to each other. The premise of this method is to have a training dataset with a marked category. The specific calculation steps are divided into three steps:

(1) Calculate the distance between the test object and all objects in the training set, where the most commonly used is *Euclidean distance* [30].
(2) Find the closest *K* objects in the distance calculated in the previous step as neighbors of the test object.
(3) Find the object with the highest frequency among the *K* objects, and its category is the category to which the test object belongs.

- Multilayer Perceptron (MLP)

**Figure 2:**
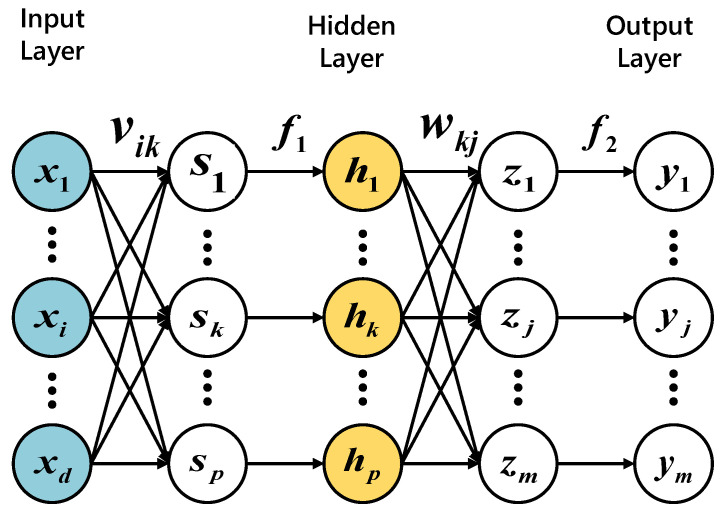
Structure of MLP.

*Multi-Layer Perceptron (MLP)* [29] is a back-propagation neural network, whose structure contains three layers: input layer, hidden layer and output layer. Each layer will be connected to the next layer, using backward pass technology to achieve supervised learning. The MLP structure is shown in Figure 2, where there are *n* pieces of input data, each piece of data corresponds to *m* output values, and only one layer of the hidden layer is set as *p* hidden nodes. Figure 2 is the structure of MLP, which shows that the value from the input layer to the hidden layer is *s_k_*, i.e., the weighted linear sum of the input values, and *v_ik_* is the weight of the *i-th* input to the *k-th* hidden node. After the activation function *f*_1_, the hidden layer node outputs *h_k_*. The value from the hidden layer to the output layer is *z_j_, j* = 1, … *m*; *w_kj_* is the weight of the *k-th* hidden node outputting to the *j-th* output value, and the output *y_j_* is obtained through the activation function *f*_2_. In short, MLP learns based on the perceptron, and changes the connection weight each time after processing the data to reduce the amount of errors between the output and the predicted results.

- Random Forest (RF)

*Random Forest (RF)* [31] is a combined learning algorithm based on decision tree classifiers. Its principle is to use a random method to build a forest, combined with multiple *CART (Classification and Regression Tree)* [32], which uses the GINI algorithm for decision-making trees to form a combined prediction model. Each tree randomly selects observations and variables to construct a classifier, and obtains the final result through voting, and then combines learning through the *Bagging algorithm* [33]. Finally, it randomly selects variables to split when CART grows. Due to its parallel computing characteristics, Random Forest performs well in both small and large datasets.

- J48 Decision Tree (J48DT)

*J48 Decision Tree (J48 DT)* [34] is an excellent improvement of the *ID3 algorithm* [32], which uses the gain ratio of attributes to overcome the problem of information gaining regularization. In the calculation process, it is also necessary to calculate the split information value of the attribute, and it performs pruning during the tree construction to avoid over-fitting. Among them, the calculation method of the split information value is:

(2) $${SplitInfo}_{A}(S)\;=\;-\sum_{j=1}^{v}\frac{\left|S_{j}\right|}{\left|S\right|}\times log_{2}\left(\frac{\left|S_{j}\right|}{\left|S\right|}\right).$$

The value of split information is used for the profit ratio, where the calculation method of the profit ratio is:

(3) $$GainRatio(A)\;=\;\frac{Gain\left(S,A\right)}{SplitInfo_{A}\left(S\right)}.$$

Note that it considers the number of branches with different characteristics. After obtaining the profit ratio, we select the maximum information profit as the feature.

- Recurrent Neural Network (RNN)

**Figure 3:**
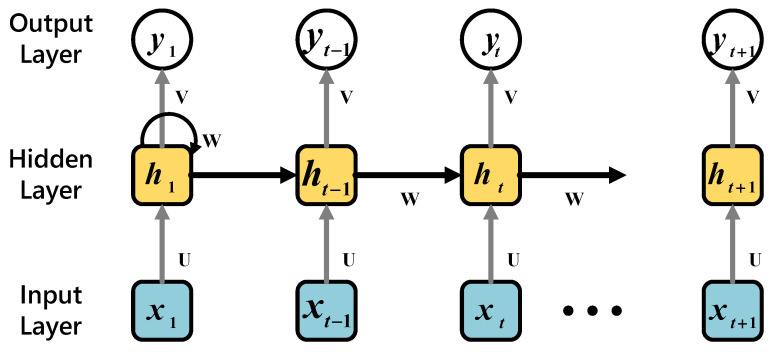
RNN model.

*Recurrent Neural Networks (RNN)* [35] can deal with time series problems. Since it considers the previous relationship, it is used as the input for the next step to maintain the state from the previous iteration to the next iteration to realize a chain of repeating modules of a neural network. In the standard RNN, the repeating module has a very simple structure, as shown in Figure 3. The specific operation steps are as follows: (1) Input *x_t_*. (2) Calculate the current state *h_t_* by using the input and the state at the previous moment. (3) The current state becomes the previous state of the next step *h_t_*_−1_. (4) Perform the above steps several times depending on assignment decision. (5) The final state is used to calculate the output *y_t_*. (6) Compare the output with the real label and get the error. (7) The error is updated through back-propagation, and the training is completed.

- Long Short-Term Memory (LSTM)

**Figure 4:**
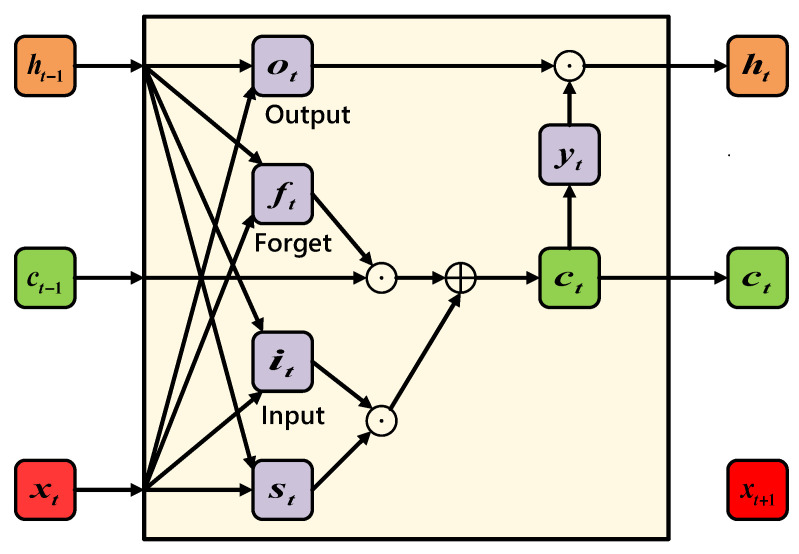
LSTM structure.

The *Long Short-Term Memory (LSTM)* model [36] also has the form of a chain of repeated modules of neural networks, as shown in Figure 4. The performance of LSTM is usually better than RNN or Hidden Markov Model. In order to minimize the training error, *gradient descent* can be used to modify the weight of each time based on the error. When the LSTM block is set, the error is also calculated backwards from *Output* to each *Gate* in the *Input* stage, until this value is filtered out. Therefore, the normal backward pass neuron is an effective method for training LSTM blocks to remember long-term values. Compared with RNN, LSTM has additional *C_t_* for memory purpose. 

By implementing of the above machine learning methods, the benefits will be evaluated in the next section.

## 5. Performance Evaluation

In this section, we use the ARAS dataset [24] for training and testing experiments, which contains the real data collected for multi-resident environments, including two houses (i.e., House A and House B) and each house has two residents, recording for 30 days of home activities. We evaluate and compare the performance of the proposed machine learning models by the performance metrics based on *confusion matrix*, shown in Table 10. They are precision, recall, accuracy, and F1-socre, where *Precision =* $\frac{TP}{\left(TP+FP\right)}$; *Recall =* $\frac{TP}{\left(TP+FN\right)}$; *Accuracy =* $\frac{TP+TN}{TP+TN+FP+FN}$; *F1-Score =* $\frac{2\times Precision*Recall}{Precision+\;Recall}$. In addition, we use *10-Fold Cross-Validation* [37] to validate all the models.

### 5.1. Precision 

**Figure 5:**
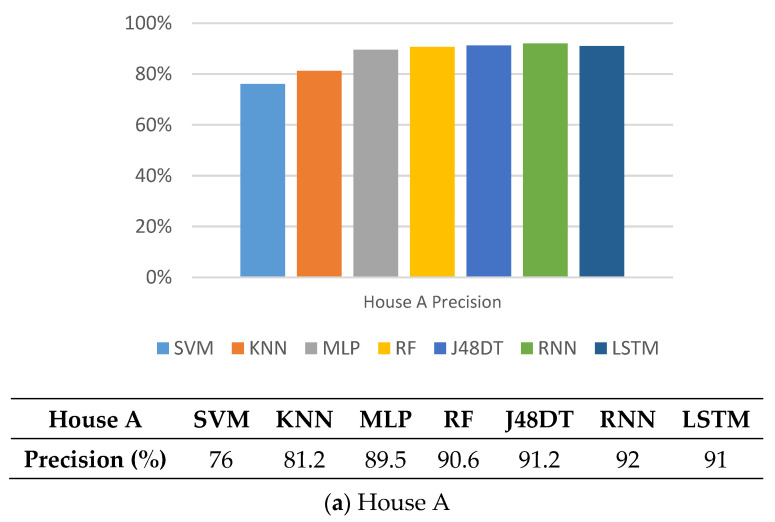

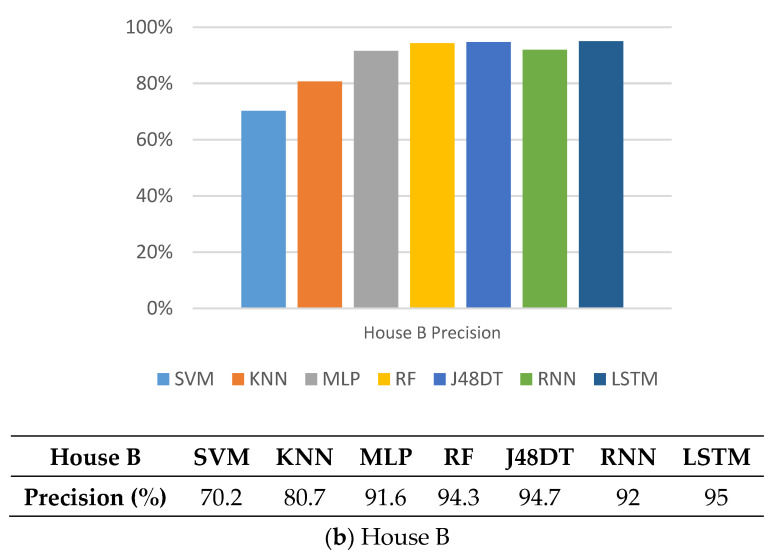
Comparison on precision of different methods.

First, we investigate the performance of different methods on precision. From Figure 5, we can see that the precision of SVM and KNN is quite low. Since KNN uses the distance between the unit to be classified and the neighboring unit as the weight for classification, once the feature types of the neighboring unit are fewer, it will cause classification errors. For SVM, since it needs to establish an accurate hyper-plane, if the sampling data is less, it results in low precision. Note that other methods perform well. Their precision values range between 89% and 95%. RNN and LSTM perform the best in House A and House B, respectively, due to the characteristic of repeated module chain of neural networks, which are more suitable for time-series features.

### 5.2. Recall 

**Figure 6:**
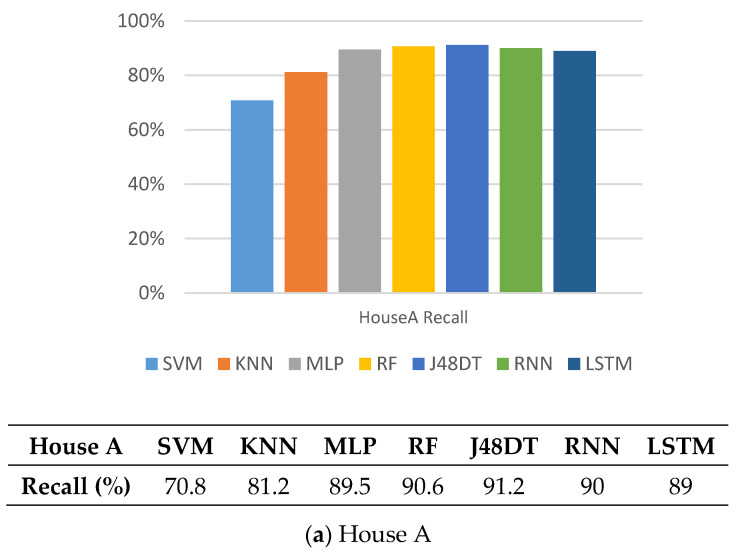

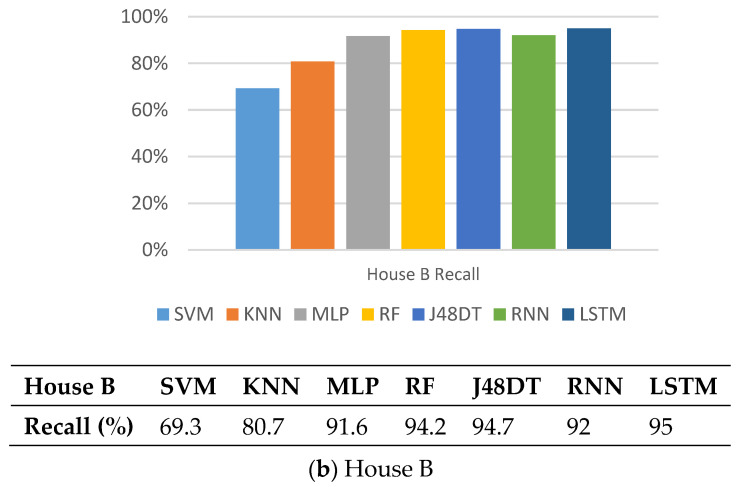
Comparison on recall of different methods.

Next, we observe the performance of different methods on recall. Similarly, Figure 6 shows that SVM and KNN perform poorly, mainly because there are fewer training features and less sampling training data. Other methods perform better. Their recall values reach up to 91% (in House A), and 95% (in House B), respectively. J48DT and LSTM perform the best in House A, and House B, respectively, because J48DT conducts pruning during tree construction to avoid over-fitting while LSTM can use the memory to remember long-term information, and thus can classify more accurately.

### 5.3. Accuracy

**Figure 7:**
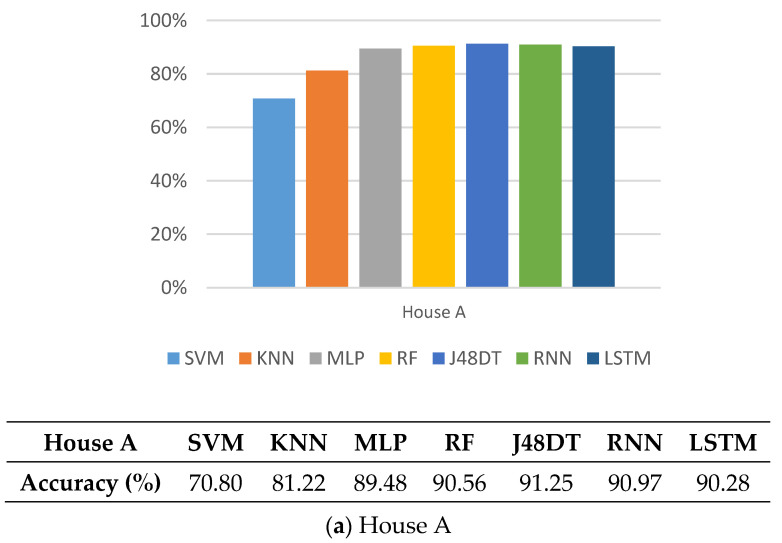

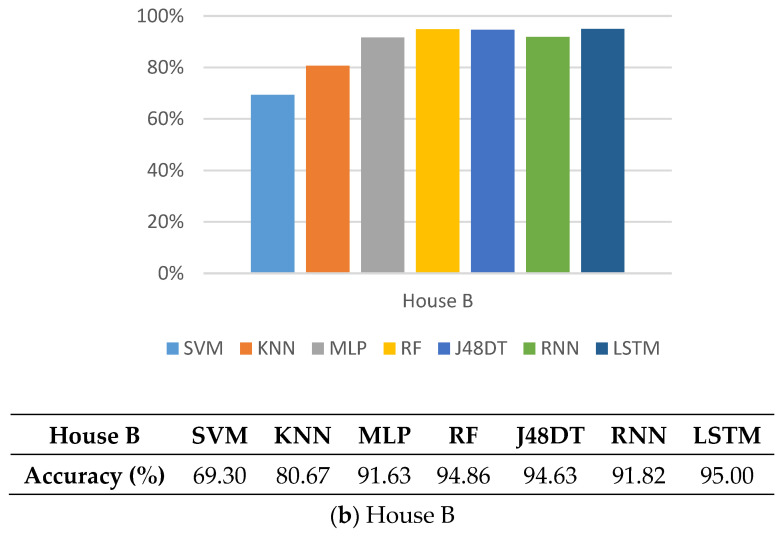
Comparison on accuracy of different methods.

Furthermore, we compare the accuracy of different methods. Similarly, Figure 7 shows that SVM and KNN have poor performance, while other methods have better accuracy (between 89.4% and 95%). Note that J48DT and LSTM perform the best. Their accuracy values reach up to 91.25% and 95%, in House A, and House B, respectively.

### 5.4. F1-Score 

**Figure 8:**
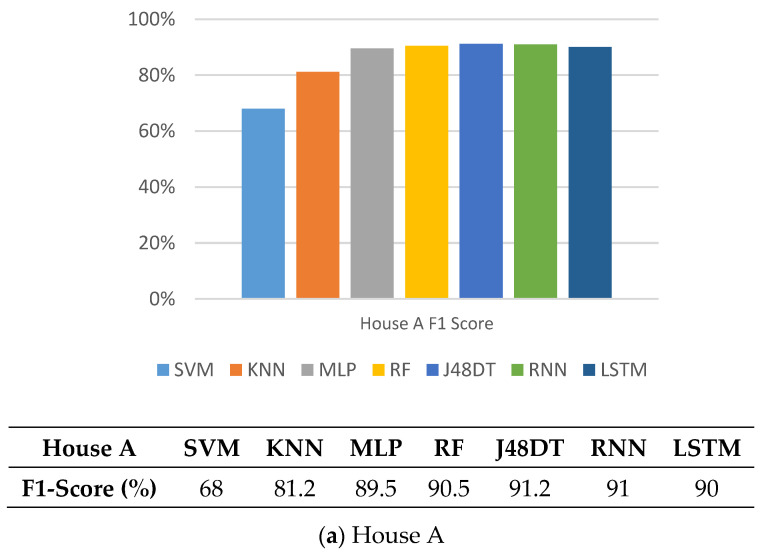

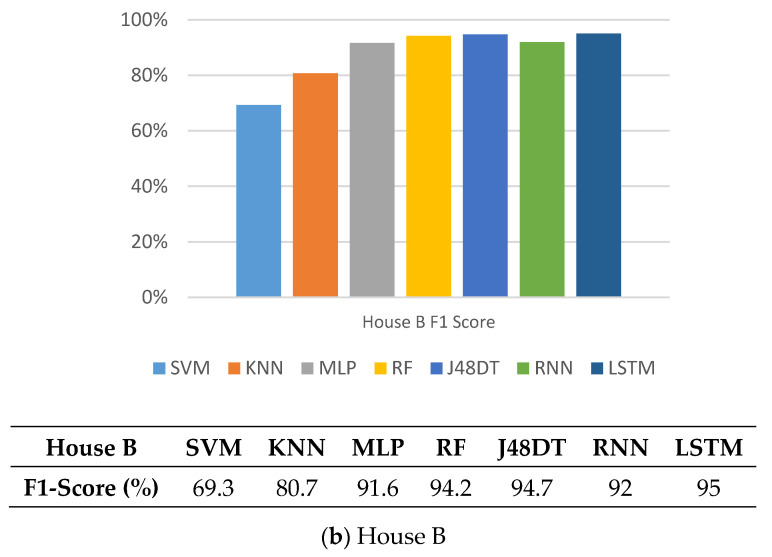
Comparison on F1-Score of different methods.

Here, we compare F1-Score of different methods, which can be used to judge the quality of the models. As shown in Figure 8, since F1-Score combines with the values of accuracy and recall as evaluation indicators, the trend of SVM and KNN will be consistent with the previous figures. Similarly, other methods performed better, whose values are between 89.5% and 95%. Note that J48DT and LSTM perform the best and their values reach up to 91.2% and 95%, in House A, and House B, respectively.

### 5.5. RMSE

**Figure 9:**
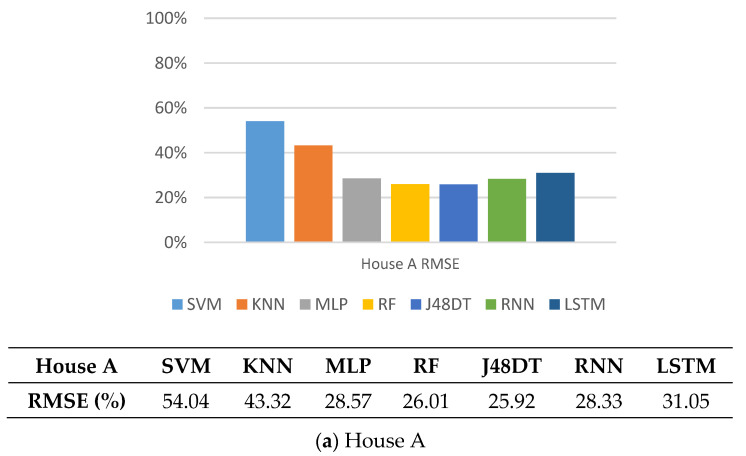

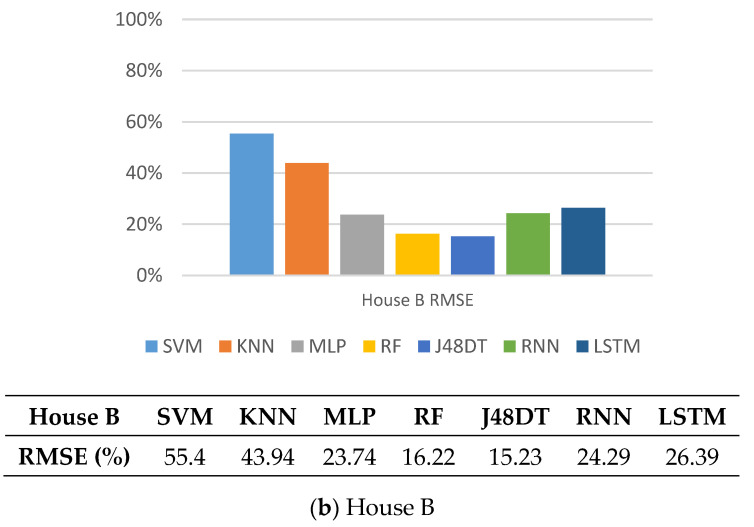
Comparison on RMSE of different methods.

*RMSE (Root Mean Square Error)* is used to measure of the deviation between the observed value and the true value. It is sensitive to the extra large or small errors in the measurement. A method with a smaller RMSE value, it can be the better learning model. Thus, if the RMSE value is close to 0, it means that the model is better than the method of predicting with the mean value. In Figure 9, we can see that the RMSE value of KNN and RF are relatively high. In relation to the results from confusion matrix (i.e., Figure 5, Figure 6, Figure 7 and Figure 8), it can be seen that the two methods are not good enough. Contrarily, the decision-tree based models (i.e., J48DT and RF) and deep learning models (i.e., RNN and LSTM) have lower RMSE, and thus, perform better.

### 5.6. Computational Complexity 

**Figure 10:**
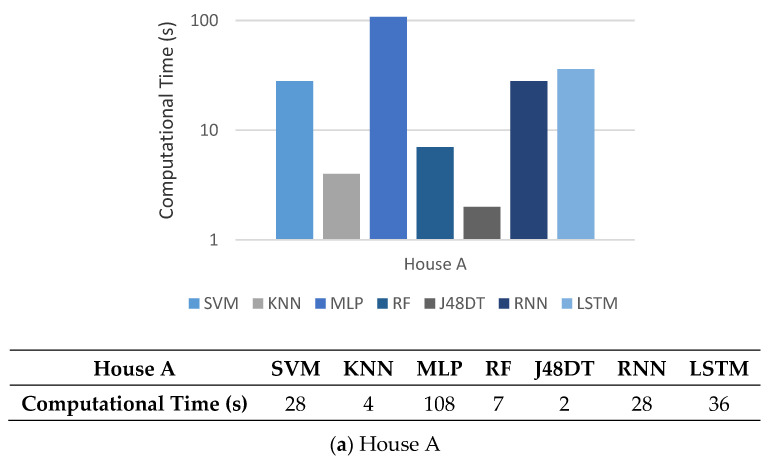

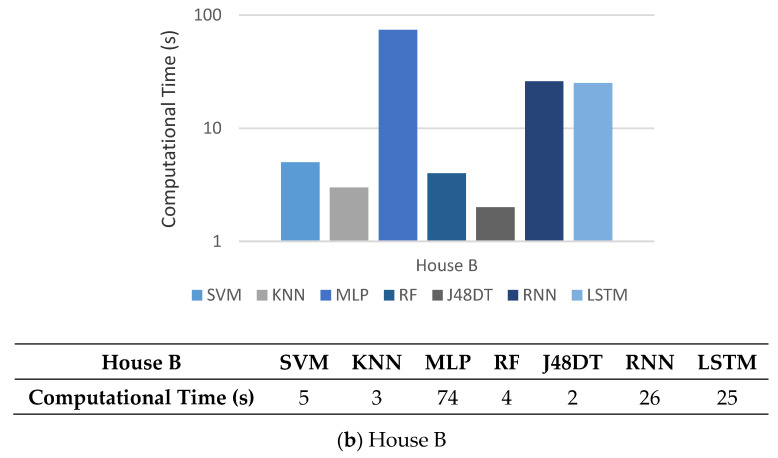
Comparison on computational complexity of different methods.

Finally, we compare the computational complexity of different methods. The computation time for training and testing is measured by the platform of ASUS D320MT with Intel Core i5-6400 CPU@ 2.7 GHz, 16 GB RAM, and NVIDIA GeForce^®^ GTX 1080Ti Graphics Card. In Figure 10, we can see that for both House A and House B, KNN and J48DT take less time than other methods. Although RNN and LSTM need more time, they can achieve more reliable precision, accuracy, and F1-score (referring to Figure 5, Figure 6, Figure 7 and Figure 8).

### 5.7. Observations

**Figure 11:**
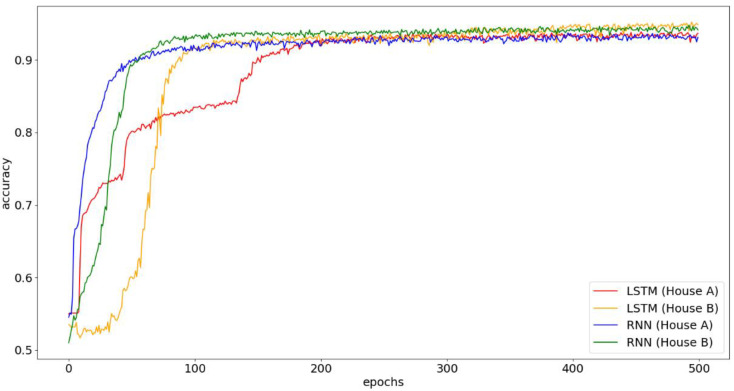
Convergence graph of RNN and LSTM.

In Figure 11, we further observe the convergence of RNN and LSTM in House A and House B on accuracy. As can be seen, the accuracy of RNN and LSTM in House A is worse than that of House B. This is because the difference in number of home activities, which are triggered by the two users in House A, is very large (i.e., one of the two users in House A often goes outside and has fewer activities, referring to Table 5), resulting in misclassification of uneven weight distribution. In addition, LSTM finally performs better than RNN in House B because the number of users’ activities is more balanced and the advantages of the memory and forgetting gate of LSTM can be enlarged to improve accuracy. Finally, we also can see that RNN converges faster than LSTM in both House A and House B, as LSTM has more parameters to be trained and updated under the same number of layers and thus converges slightly slow.

### 5.8. Discussions

Since SVM needs to find the best boundary between classification targets, if there is more data or the situation becomes more complex, its performance decreases dramatically, as shown in Figure 5, Figure 6, Figure 7 and Figure 8. Then, KNN exploits the distance between the classification target and the adjacent data as the classification weight. Therefore, once the features of adjacent units are fewer, it will cause errors. Therefore, it does not perform well in precision, recall, accuracy and F1-score (as shown in the above figures). However, its intuitive classification mechanism only needs to find a specific number of units near the target for comparison, so the training time is less, as shown in Figure 10. MLP is a multi-layer feed-forward neural network, which has better performance in precision, recall, accuracy and F1-score (referring to Figure 5, Figure 6, Figure 7 and Figure 8). However, if there are more parameters to be learned, it takes lots of time for calculation. RF and J48DT are both decision tree based algorithms, which need to find the best solution from branches. Therefore, they perform very well in precision, recall, accuracy and F1-score. Note that the activity records in House A is much more than that in House B, so the branch nodes and tree width of House A in the RF method are more than that in House B, resulting in more training time for House A than House B (referring to Figure 10). J48DT always selects the option with the highest information gain at the branch point, so it can be reduced the width of the tree and has the least calculation time. RNN is a deep-learning model based on the time dimension and has a memory function that can retain information during the training process. Therefore, it performs better than ordinary neural networks. From Figure 5, Figure 6, Figure 7 and Figure 8, we can see that it has better performance in terms of precision, recall, accuracy and F1-score. However, it also requires more training parameters and thus spends more time in training. LSTM has a long-term memory and forgetting mechanism, so it performs better in precision, recall, accuracy and F1-score than that of RNN. In addition, the difference in recall between House A and House B is large (i.e., 89%, and 95%, respectively), as House A has more data (i.e., 306 records) and House B has less data (i.e., 159 records). Note that the performance of the deep learning models (i.e., LSTM and RNN) is better (but slightly) than that of the decision-tree based learning (i.e., J48DT and RF) as the dataset is scarce and the scale is slightly small (i.e., only 30 days of records), and thus, LSTM and RNN may limitedly elaborate the sequential activity features with the memory mechanism and forgetting gate. Contrarily, J48DT always selects the option with the highest information gain at the branch node especially when the dataset is not very large and then performs pruning during the tree construction process to avoid over-fitting. Thus, J48DT can perform well.

## 6. Conclusions

In this paper, we have addressed the problem of multi-resident activity recognition. Based on the activity dependent features and user historical behaviors, we have implemented and evaluated five traditional machine learning methods and two deep learning techniques for training and testing, including SVM, KNN, MLP, J48DT, RF, RNN and LSTM. According to the experimental results with real datasets, the performances of J48DT and LSTM are the best, whose accuracy values reach up to 91%, and 95%, respectively, in different houses. While, the performances of SVM and KNN are the worst, even lower than 69%. For the future work, we will continue to investigate more powerful learning technologies, such as reinforcement learning, and also compare them with more presentative algorithms, in order to provide more valuable experiences for the development of the elderly personal care and further expand the application scope.

## Figures and Tables

**Table 1:** Comparison of the literature for recognition.

Reference	Aim	Proposed Methods	Pros	Cons
[23]	To demonstrate how human motions can be detected in a quasi-real-time scenario.	Non-invasive machine learning algorithm	(1) Less expensive (2) Requires fewer resources	Limited activity consideration.
[9]	To provide monitoring, recording and identification of human daily activities through cameras	To identify the daily activities of the elderly based on the characteristics of skeleton and joint.	Enriching the data diversity	Face privacy concern.
[10]	To introduce vision-based human action recognition	To quantify and compare the vision-based methods	Providing an overview and summarize the challenges	Face privacy concern
[11]	To design a privacy-preserving and computationally efficient framework	Using mobile video analytics based on convolutional neural network model	Less data processing time	Not focusing on identification
[12]	To design wearable-sensors based healthcare system for human activity recognition.	IoT and blockchain based data acquisition, transmission and data encryption modules	Alarming feature	Needing to wear sensors
[13]	To use multiple wearable sensors for activity recognition	To exploit a probabilistic model based on Hidden Markov Models	Useful for automatic ergonomic evaluation for industrial applications	Needing to wear sensors
[14]	To recognize activity based on the localization of wearable sensors	Using a two-stream Convolutional Neural Networks	Simultaneous recognition of both human activity and sensor location	Needing to wear sensors
[15]	To make wearable devices be the ubiquitous platform for data acquisition and analysis	To obtain body action through inertial sensors of wearable devices and mobile phones	Easy to get and implement	Needing to wear sensors
[16]	To recognize activity under the interference of passengers	Based on multiple WiFi signal information	Increasing the diversity of fusion data	Focusing on single-person (i.e., the driver) activity recognition.

**Table 2:** Types of home activity [24].

Num.	Activity	Num.	Activity
1	Idle	15	Toileting
2	Going out	16	Napping
3	Preparing breakfast	17	Using Internet
4	Having breakfast	18	Reading book
5	Preparing lunch	19	Laundry
6	Having lunch	20	Shaving
7	Preparing dinner	21	Brushing teeth
8	Having dinner	22	Talking on the phone
9	Washing dishes	23	Listening to music
10	Having snack	24	Cleaning
11	Sleeping	25	Chat
12	Watching TV	26	Having guest
13	Studying	27	Changing clothes
14	Having shower		

**Table 3:** Examples of historical activities and unknown user list.

*Historical Activity List* *(A_i_, T_i_, U_i_)*	
*A_1_*	(12, 17, 22, 12, 15, 17, 21, 15, 17, 11, 11, 20, 21, 15, 27, 2, 1, 12, 3, 4, 15, 17, 21, 13, 22, …)
*T_1_*	(00:00:01, 00:00:01, 00:09:03, 00:14:05, 00:43:42, 00:50:02, 00:56:18, 01:00:00, 01:04:12, …)
*U_1_*	(user_1_, user_2_, user_1_, user_1_, user_2_, user_2_, user_1_, user_1_, user_1_, …)
*A_2_*	(17, 2, 10, 17, 10, 12, 13, 21, …)
*T_2_*	(00:00:01, 00:00:01, 00:0042, 00:06:46, 00:19:40, 00:23:22, 01:07:55, 02:32:28, …)
*U_2_*	(user_1_, user_2_, user_1_, user_1_, user_1_, user_1_, user_1_, user_1_, …)
*A_3_*	(10,10,17,12,25,12,21,17,15, …)
*T_3_*	(00:00:01, 00:00:01, 00:01:38, 00:18:49, 00:29:01, 00:47:54, 01:29:31, 01:30:35, 01:36:05, …)
*U_3_*	(user_1_, user_2_, user_2_, user_1_, user_1_, user_1_, user_2_, user_1_, user_2_, …)
*A_4_*	(12, 17, 21, 17, 11, 22, 17, 21, 11, …)
*T_4_*	(00:00:01, 00:00:01, 00:05:11, 00:06:13, 00:08:28, 00:12:02, 00:23:45, 01:03:08, 01:12:26, …)
*U_4_*	(user_1_, user_2_, user_2_, user_1_, user_2_, user_1_, user_1_, user_1_, user_1_, …)
*A_5_*	(22, 2, 12, 17, 21, 11, 25, 25, 25, 27, …)
*T_5_*	(00:00:01, 00:00:01, 00:10:06, 01:20:22, 01:44:29, 01:48:57, 09:16:17, 09:16:36, 09:27:18, …)
*U_5_*	(user_1_, user_2_, user_1_, user_1_, user_1_, user_1_, user_2_, user_1_, user_2_, …)
***Unlabeled Data (A_x_, T_x_, U_x_)***	
*A_x_*	(*12, 2, 17, 15, 21, 11, 12, 1, 3, 17, 3, 2, 4, 22, 4, 9, 15, 21, 12, 17, 15, 14, 17, 5, 22, 5, 6, 26, 6, 9,* …)
*T_x_*	(00:00:01, 00:00:01, 00:01:02, 00:06:01, 00:52:04, 01:15:03, 01:55:20, 02:22:26, 02:54:54, …)
*U_x_*	(user_1_, user_2_, user_1_, ?, ?, ?, ?, user_1_, ?, ?, ?, …)

**Table 4:** Notations used in the paper.

Notation	Definition
*A_i_*	Activity sequence of day *i*
*U_i_*	User sequence of day *i*
*T_i_*	Activity occurrence time of day *i*
*A_x_*	Activity sequence of unknown user
*T_x_*	Activity occurrence time (corresponding to *A_x_*)
*U_x_*	Unknown user sequence (corresponding to *A_x_*)
μ	Average value
σ	Standard deviation
*x_d_*	The value of the input layer
*s_k_*	The value from the input layer to the hidden layer
*v_ik_*	The weight of the *i-th* input to the *k-th* hidden node
*z_j_*	The value from the hidden layer to the output layer
*w_kj_*	The weight of the *k-th* hidden node outputting to the *j-th* output value
*y_j_*	The *j-th* output value
*h_t_*	The *t-th* hidden layer
*f_t_*	The *t-th* forgetting gate in LSTM model
*C_t_*	Current memory data
*O_t_*	The output of the *t-th* hidden layer
TP	True Positive
TN	True Negative
FP	False Positive
FN	False Negative

**Table 5:** The detailed attributes of the ARAS dataset [24].

Attribute	House A	House B
Num. of residents	2 males (both aged 25)	Married couple (age average 34)
Size of the house	50 m^2^	90 m^2^
House information	One bedroom, one living room, one kitchen, one bathroom	Two bedrooms, one living room, one kitchen, one bathroom.
Num. of ambient sensors	20 of 7 different types	20 of 6 different types
Duration	30 days	30 days
Num. of activities	27	27
Num. of data records (user_1_: user_2_)	1594:1288	1180:1021

**Table 6:** Combined Activities.

Num.	Activity	Num.	Activity
1	Idle	13	Using Internet
2	Going out	14	Reading book
3	Cooking	15	Laundry
4	Eating	16	Shaving
5	Washing dishes	17	Brushing teeth
6	Having snack	18	Talking on the phone
7	Sleeping	19	Listening to music
8	Watching TV	20	Cleaning
9	Studying	21	Chat
10	Having shower	22	Having guest
11	Toileting	23	Changing clothes
12	Napping		

**Table 7:** Examples of multi-label activity data.

ID	Day	Chat	Clean	Cloth	Cook	Toilet	TV	…	User
1	1	0	0	0	0	0	1	…	1
2	1	0	0	0	0	0	0	…	1
3	1	0	0	0	0	1	0	…	2
4	1	0	0	0	0	0	0	…	2
5	1	0	0	0	0	0	0	…	1

**Table 8:** Results of one-hot encoding.

ID	Day	Chat	Clean	Cloth	Cook	…	Toilet	TV	User_1_	User_2_
1	1	0	0	0	0	…	0	1	1	0
2	1	0	0	0	0	…	0	0	1	0
3	1	0	0	0	0	…	1	0	0	1
4	1	0	0	0	0	…	0	0	0	1
5	1	0	0	0	0	…	0	0	1	0
…	…	…	…	…	…	…	…	…	…	…
2876	30	0	0	1	0	…	0	0	1	0
2877	30	0	0	0	0	…	0	0	1	0
2878	30	0	0	0	0	…	0	0	1	0
2879	30	0	0	0	0	…	0	1	1	0
2880	30	0	0	0	0	…	0	0	0	1

**Table 9:** Examples of the filtered activity features.

ID	Day	uniqueID	Times	Duration	Period	Time2	Ratio	User_1_	User_2_
1	1	1	3	0.150	1	1	0.12	1	0
2	1	2	5	0.083	1	1	0.04	1	0
3	1	3	3	0.116	1	1	0.02	0	1
4	1	4	5	0.716	1	1	0.03	0	1
5	1	5	3	0.066	1	1	0.01	1	0

**Table 10:** Confusion matrix.

Actual Class\Predicted Class	Positive	Negative
Positive	TP	FN
Negative	FP	TN

## Data Availability

The data used to support the findings of this study are included within the article.
